# Molecular analysis of ex-vivo CD133+ GBM cells revealed a common invasive and angiogenic profile but different proliferative signatures among high grade gliomas

**DOI:** 10.1186/1471-2407-10-454

**Published:** 2010-08-24

**Authors:** Juan L Garcia, Maria Perez-Caro, Juan A Gomez-Moreta, Francisco Gonzalez, Javier Ortiz, Oscar Blanco, Magdalena Sancho, Jesus M Hernandez-Rivas, Rogelio Gonzalez-Sarmiento, Manuel Sanchez-Martin

**Affiliations:** 1 Instituto de Estudios de Salud de Castilla y León (IESCyL), Spain; 2 IBMCC, Centro de Investigación del Cáncer (USAL/CSIC), Salamanca; 3 OncoStem Pharma S.L, Spain; 4 Department of Neurosurgery, Hospital Universitario de Salamanca, Spain; 5 Department of Pathology, Hospital Universitario de Salamanca, Spain; 6 Department of Haematology, Hospital Universitario de Salamanca, Spain; 7 Department of Medicine, University of Salamanca, Spain; 8 Genetically Engineered Mouse Facility, SEA, University of Salamanca, Spain

## Abstract

**Background:**

Gliomas are the most common type of primary brain tumours, and in this group glioblastomas (GBMs) are the higher-grade gliomas with fast progression and unfortunate prognosis. Two major aspects of glioma biology that contributes to its awful prognosis are the formation of new blood vessels through the process of angiogenesis and the invasion of glioma cells. Despite of advances, two-year survival for GBM patients with optimal therapy is less than 30%. Even in those patients with low-grade gliomas, that imply a moderately good prognosis, treatment is almost never curative. Recent studies have demonstrated the existence of a small fraction of glioma cells with characteristics of neural stem cells which are able to grow *in vitro *forming neurospheres and that can be isolated *in vivo *using surface markers such as CD133. The aim of this study was to define the molecular signature of GBM cells expressing CD133 in comparison with non expressing CD133 cells. This molecular classification could lead to the finding of new potential therapeutic targets for the rationale treatment of high grade GBM.

**Methods:**

Eight fresh, primary and non cultured GBMs were used in order to study the gene expression signatures from its CD133 positive and negative populations isolated by FACS-sorting. Dataset was generated with Affymetrix U133 Plus 2 arrays and analysed using the software of the Affymetrix Expression Console. In addition, genomic analysis of these tumours was carried out by CGH arrays, FISH studies and MLPA;

**Results:**

Gene expression analysis of CD133+ vs. CD133- cell population from each tumour showed that CD133+ cells presented common characteristics in all glioblastoma samples (up-regulation of genes involved in angiogenesis, permeability and down-regulation of genes implicated in cell assembly, neural cell organization and neurological disorders). Furthermore, unsupervised clustering of gene expression led us to distinguish between two groups of samples: those discriminated by tumour location and, the most importantly, the group discriminated by their proliferative potential;

**Conclusions:**

Primary glioblastomas could be sub-classified according to the properties of their CD133+ cells. The molecular characterization of these potential stem cell populations could be critical to find new therapeutic targets and to develop an effective therapy for these tumours with very dismal prognosis.

## Background

The cancer relapse and mortality rate suggests that current therapies do not eradicate all malignant cells. In this sense, there is increasing evidence that many types of cancer contain their own stem cells: cancer stem cells (CSCs), which are characterized by their self-renewing capacity and differentiation ability [1]. The study of haematological disorders shed light on the relationship between cancer and stem cell compartments, and the mechanisms by which CSCs might appear and change during the progression of the disease [2,3]. However, the evidence for the existence of CSCs in solid tumours has been more difficult to find because of the lack of specific cell surface markers. During the last years, different cancer cell subpopulations from selected types of human solid cancers have been identified (breast [4], brain [5-7], colon or colo-rectal [8-10], head and neck [11] and pancreatic cancer [12]). These authors, through the use of cell culture, FACS and/or MACS methods, have been able to identify different cell populations within the tumour showing hallmarks of stem cells. This stem cell potential, including self-renewal and lineage capacity, was demonstrated by serial transplantation experiments in animal models. Specifically, the investigation of solid tumour stem cells has gained momentum particularly in the area of gliomas, the most common type of brain tumours. In this group, glioblastoma multiforme is the highest-grade glioma [GBM; grade IV] and is manifested by morphological, genetic and phenotypic heterogeneity [13-15]. Two major aspects of glioma biology that contributes to its awful prognosis are the formation of new blood vessels through the process of angiogenesis and the invasion of glioma cells, the hallmarks of GBM [16]. In addition, these abnormal blood vessels have also been shown to create a vascular niche that houses glioma stem cells [17].

Despite of the recent advances, two-year survival for GBM patients with the most favourable treatment is less than 30%. Even in those patients with low-grade gliomas therapy is almost never curative. Recent studies have confirmed the existence of a small portion of glioma cells with characteristics of neural stem cells [1]. In general, this fraction is characterized by its neurosphere-forming ability, its strikingly increased drug resistance, and finally, by its ability to express surface markers that are oftenly used for their FACS-based isolation [5,6]. With the implantation during this last decade of the NS forming assay as a robust method for the isolation of neural stem cells [18], it has become widely accepted that adult mammalian brain harbours a pool of NSCs responsible for the persistent neurogenesis, seen in limited adult brain regions, such as the sub-ventricular zone, olfactory bulb and hippocampal dentate gyrus [19].

However, it should be borne in mind that the NS assay is not the most suited source of primary adult stem cells for transcriptomic analysis since cells are selected based on its *in vitro *proliferation capacity in the presence of cytokines and growth factors in their cell cultures such as EGF and FGF.

At the end of twenty century, two independent laboratories could identify and isolate human central nervous system stem cells using antibodies against CD133 [20,21]. This protein, named prominin, identifies a subset of human foetal brain cells distinct to human haematopoietic stem cells, which are also CD133+ but are also CD34-bright [22]. This subset of human CD133+ fetal brain cells is capable of neurosphere initiation, self-renewal, and multilineage differentiation at the single-cell level [20]. The CD133+ cells can differentiate *in vitro *to neurons and glial cells, and their transplantation into the lateral ventricles of newborn NOD-SCID mouse brains resulted in specific engraftment in numerous sites of the brain [20,21,23].

The CD133 marker is a five-transmembrane protein which is expressed in different type of progenitors as human fetal brain cells or human hematopoietic stem cells [20-22]. In brain tumours the proportion of these CD133+ cells represent a minority of the tumour cell population and are also capable to initiate tumour formation *in vivo*. Although it have also been reported that a proportion of these tumours could be maintained by CD133- cells [24], there are several evidences showing how this small fraction of CSC which forming NS, can also be isolated using CD133 as a selection marker [6].

In the present study, we have analysed thoroughly the molecular signature of eight fresh primary GBMs focused on its CD133 positive and negative cells. Importantly, all tumours were studied before any treatment of the patient and without previous tumour cell culturing. In addition to the expression analysis of the FACS-sorted cells, we have also performed genome-wide analysis by CGH-arrays, FISH studies at PTEN and EGFR loci, and MLPA at the MGMT promoter. The results obtained concluded that the gene expression signature of CD133+ discriminate common genes to all samples involved in two main characteristic pathways deregulated in GBMs, angiogenesis and invasiveness. However, CD133+ gene expression profile also allowed distinguishing between two different GBM subtypes in higher or lowering proliferative tumours. The molecular biology and the expression signature of these CD133+ cells that drive and support the tumour growth will shed light on the development of fresh and specific treatment strategies.

## Methods

### Samples, flow cytometry and sorting assays

**Figure 1:**
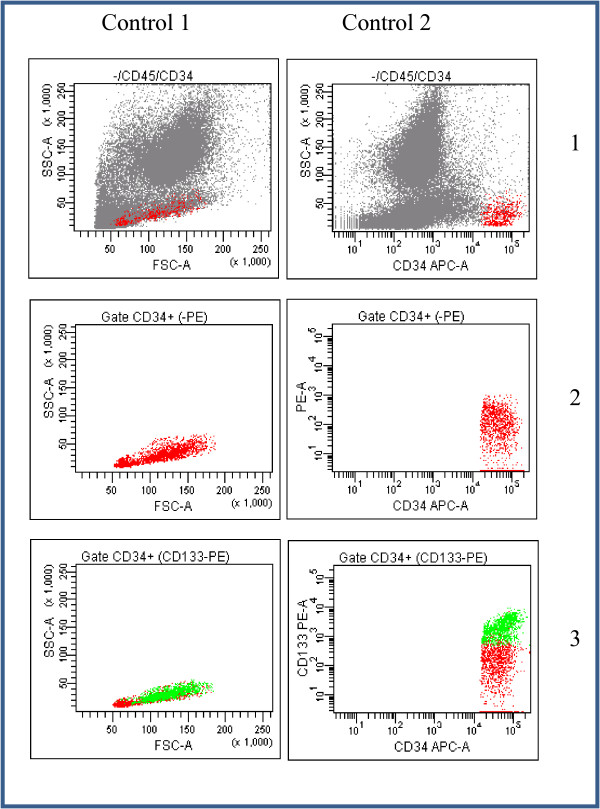
**FACS sorting of glioblastoma cells using CD133 and CD34 antibodies**. Control samples from human bone marrows incubated with CD133 antibody. 1: Total cellularity; 2: Gate CD34 without CD133-PE; 3: Gate CD34 with CD133-PE.

Fresh tumours from eight patients affected of primary GBM without any previous treatment were collected (clinic and pathologic features are summarized in Table 1). Patients' diagnostic were confirmed by the Pathology Facility from the University Hospital of Salamanca, Spain. At the surgical extraction moment, a vast proportion of each tumour was processed to isolate the CD133+ and CD133- cells without previous cell culture. Single-cell suspensions were prepared from individual tumours by standard procedures. We decided to undergo a mechanical disaggregation of tumour samples due to the softness of brain samples, avoiding enzymatic stress that could change the cell surface and even their gene expression. Briefly, tumours were carefully sliced and forced through a 70 μm single-cell filter into the Ca^++^/Mg^++ ^free phosphate-buffered saline by applying gentle pressure using the piston of a disposable plastic syringe, All single-cells were used for staining. Cells were immunophenotyped using human CD133/2 (293C3) phycoerythrin conjugated antibody (MACS, Miltenyibiotec). Mature red cells were depleted by hypotonic lysis solution (0.38% ammonium chloride for 15 minutes on ice) before staining. Cells suspended in Ca^++^/Mg^++ ^free phosphate-buffered saline supplemented with 1% fetal calf serum were labelled with this antibody (approximately 1 μg/10^6 ^cells) for 30 minutes on ice. Cell fluorescence was analyzed and sorted using the FACS Aria sorter (Becton Dickinson, New Jersey, USA). CD133 antibody was tested previously in human bone marrow (BM) cells in which CD133 positive cells were described before (Figure 1). BM cells were incubated with CD133 and CD34 antibodies (Pharmingen), sustained in studies that demonstrate that antibodies against CD133 also identified a subset of CD34^bright ^BM hematopoietic stem cells [22]. Cell viability was assessed by propidium iodide exclusion (5 μg/mL; Sigma) using flow cytometry.

**Table 1:** Clinical characteristics, MGMT promoter methylation and FISH analysis of eight primary GBMs.

GBM sample	Sex	Age years	[M1]Number CD133 (%)	Stage	Tumour	Tumour Location	Resection	DFP months	Survival Days[M2]	Radiotherapy	Chemotherapy	Response	MGMT Methylation	EFGR (% cells)	PTEN (% cells)
**G2**	M	73	2300 (0'5%)	Death	GBM	Temporal	Total	9	398	Cranial+Boost	TMZ	CR	No met	Amplification (74%)	del (46%)
**G4**	M	70	37000 (3'2%)	Death	GBM	Temporal	Total	5	479	Cranial	-	NR	Met	Polysomy (61%)	del (86%)
**G5**	F	68	6000 (1'5%)	Alive	GBM	Frontal	subtotal	8	630	Cranial+Boost	TMZ	PR	Met	Polysomy (55%)	del (64%)
**G6**	M	65	6000 (1'6%)	Death	GBM	Multifoci	subtotal	5,5	410	Cranial	-	CR	No Met	Polysomy (47%)	Normal
**G7**	M	59	4800 (0'6%)	Death	GBM	Frontal	Total	3	192	Cranial	TMZ	P	No met	Amplification (54%)	del (40%)
**G8**	M	65	6000 (0'7%)	Alive	GBM	Temporal	Total	4,5	166	Cranial	TMZ	P	Met	Normal	del(70%)
**G9**	M	69	2900 (0'1%)	Death	GBM	Parietal	subtotal	2	108	Cranial+Boost	-	P	nd	Normal	Normal
**G11**	F	76	13000 (1'7%)	Death	GBM	Tempo-parietal	Total	6	377	Cranial+Boost	TMZ	NR	No met	Amplification (72%)	del (79%)

Radiation therapy was administrated in all cases, cranial radiation (four cases) or cranial + Boost radiation. A total dose of 60 GY was administrated 5 days per week during 6 weeks. Additionally, in four cases, Temozolomide was administrated daily beginning on the first day of radiation.

M: Male; F: Female; CR: complete response (no residual disease can be identified on clinical examination); DFP: disease free period (time of period from surgery to detection of signs of disease); PR: partial response (reduction of disease by 30% or more on clinical examination); P: progression (disease has increased in size or number on treatment); NR: no response; nd: no date; TMZ: Temozolomide

### Expression arrays

We studied a dataset generated with Affymetrix U133 Plus 2 arrays (Affymetrix, Santa Clara, CA, USA) in 8 gliomas. Results from this expression analysis have been deposited at GEO [25] with accession number GSE18015.

Isolated cells (CD133+ and CD133- from each tumour) using sorting methods were collected in separated vials containing RNA Later (Qiagen, Chatsworth, CA, USA). Total RNA was extracted from CD133+ and CD133- sorted cells using Trizol (Invitrogen, Carlsbard, CA) making a total of 16 samples (8 positives and 8 negatives). The integrity of the RNA was confirmed with the Agilent Bioanalyzer 2100 using the RNA 6000 Pico kit (Agilent). We used the GeneChip^® ^Expression 3' Amplification Two-Cycle Target Labelling kit (Affymetrix, Santa Clara, CA, USA) to label the RNA following the manufacturer protocol. The cRNA was hybridized to Affymetrix Human U133 Plus 2 arrays according to the manufacturer protocol. Briefly, double-stranded cDNA was synthesized routinely from less than 1 microgram of total RNA primed with a poly-(dT) -T7 oligonucleotide. The cDNA was used in an *in vitro *transcription reaction in the presence of T7 RNA polymerase and biotin-labelled modified nucleotides during 16 hours at 37°C. Biotinylated cRNA was purified and then fragmented (35-200 nucleotides), together with hybridization controls and hybridized to the microarrays for 16 h at 45°C. Using the GeneChip Fluidics Station 450 (Affymetrix), the biotin-labelled cRNA was revealed by successive reactions with streptavidin R-phycoerythrin conjugate, biotinylated anti-streptavidin antibody and streptavidin R-phycoerythrin conjugate. The arrays were finally scanned in an Affymetrix GeneChip Scanner 7G Plus.

Preliminary data analysis was conducted using the software of the Affymetrix Expression Console from AGCC suite (Affymetrix GeneChip Command Console, version 1.1) following the statistical procedures described in the Affymetrix: Expression Console User Guide, selecting the 3' Expression Analysis guidelines for MAS5 and PLIER algorithms in two independent steps. MAS5 calculated the present call index for each of the 54,675 probe sets on the chip (settings used were standard for the HG U133 Plus 2 array: alpha1 = 0.04, alpha2 = 0.06, Tau = 0.015, TGT = 500). This present call index was used to select 245 probe sets having Presence index through the 16 analyzed samples. PLIER algorithm was used to calculate the normalized expression values of the probe sets (using quantile normalization and PM-MM background correction methods). Statistical analysis and post-processing were performed using TIBCO Spotfire 9.1 (TIBCO Software Inc., Palo Alto CA, USA).

Multidimensional Scaling Analysis of CGH and Gene Expression data for the eight samples was performed using the multivariate statistics options of the Simfit Package (Version: 6.1.10 ^© ^W. G. Bardsley, University of Manchester, http://www.simfit.man.ac.uk/).

Network analysis was performed mapping the results on the IPA 8 knowledge database (Ingenuity Pathway Analysis).

### CGH array

DNA from each fresh-frozen sample was extracted with the standard phenol-chloroform method and normal DNA was prepared from human placenta of healthy donors. DNAs were quantified using the Nanodrop spectrophotometer. DNA quality was assessed by the 260:280 ratio and its integrity by agarose gel ethidium bromide visualization. Genomic-wide analysis of DNA copy number in each patient was performed using CGH based array. Due to the low proportion of CD133 positive cells in each tumour sample, CGH array only was performed using genomic DNA from the bulk tumour. Slides containing 3296 BACs were produced in "Centro de Investigación del Cáncer" (Salamanca, Spain). The particular bacterial artificial chromosome (BAC) and P-1 derived artificial chromosome set used to produce this array is distributed to academic institutions by the Welcome Trust Sanger Institute (Cambridge, United Kingdom) and contains targets spaced at ≈ 1 Mb density over full genome, a set of subtelomeric sequences for each chromosome arm and a few hundred of probes selected for their involvement in oncogenesis. The clone content is available in the "Cytoview" windows of the Sanger Institute mapping database site, Ensembl (http://www.ensembl.org/). According to this database, clones were ordered along the chromosomes. For the array, 10 simultaneous hybridizations of normal male versus normal female and placenta (DNA reference) was performed to define the normal variation for the log_2 _ratio. Cy5/Cy3 intensity ratios of every spot were converted into log_2 _ratios. The log_2 _ratio of each clone was normalized to the median log_2 _ratio of the ten control hybridizations, after which the median of triplicate spots was calculated. Data from two-colour hybridizations for both DNA were normalized using the corresponding GEPAS module DNMAD [26]. Regions of copy number gained and lost for the BAC array-CGH data were identified by creating sample specific thresholds. Reference copy number polymorphisms were carefully revised in all data sets. Therefore every clone on the array was compared with 'Database of Genomic Variants' (http://projects.tcag.ca/variation/) [27,28]. For unsupervised clustering analysis, we converted the relative ratio value for each BAC clone to a score of 1 (gained/amplified), 0 (no change), or -1 (lost) based data obtain by the binary segmentation method described by Olshen et al. [29] and analyzed data with Cluster and TreeView of GEPAS (Multi Experiment Viewer 4.0) based on the average linkage method with the Pearson uncentered metric correlation. Statistical evaluation was carried out using the SPSS 15.0 statistical software (Chicago, Illinois, USA). All P-values reported were two-sided and statistical significance was defined as P-values < 0.05. Complementary details on this method are summarised in Additional file 1.

### Fluorescence in situ hybridization

Dual-probe fluorescence *in situ *analysis were performed with locus-specific probes for centromere 7/EGFR gene and centromere 10/PTEN gene (Vysis, Dowerners Grove, IL). FISH studies were carried out following well-established methods [30]. Polysomies (chromosomal gains) were defined as more than 10% of nuclei containing three or more CEP signals. Specimens were considered to have an amplification of EFGR when more than 10% of CD133 negative tumour cells exhibited an EGFR/CEP7 ratio >2 or inestimable tight clusters of signals of the locus probe.

### Real-Time PCR

CD133+ and CD133- amplified RNA samples were reverse-transcribed to cDNA. PCR reactions were performed using equal amounts of cDNA as template. SYBR Green PCR Master mix (Applied Biosystems, USA) was used for template amplification using standard protocol with specific primers for each of the transcripts examined [Additional file 2: Supplemental Table S1]. Incorporation of the SYBR Green dye into PCR products was monitored in real time with an ABI PRISM 7000 sequence detection system (Applied Biosystems). SDS system software was used to convert the fluorescent data into threshold cycle (*C*_t_) at which exponential amplification of products begins. The differences in the *C*_t _values (d*C*_t_) between the transcript of interest and endogenous control (GAPDH) were used to determine the relative expression of the gene in each, adapted from [31]. qPCR was performed using specific primers to corroborate expression array result for several genes of those 245 presented probes [Additional file 3: Supplemental Figure S1].

### MS-MLPA analysis

MLPA analysis was performed using SALSA MLPA Kit ME011 and executed as described by the manufacturer (MRC-Holland) with minor modifications in order to detect MGMT promoter methylation. Briefly, 5 μl of each tumour DNA was denatured and subsequently cooled down to 25°C. After addition of the probe mix, the sample was denatured and probes were allowed to hybridize. Sample was ligated with or without HhaI digestion, resulting in ligation of the only digested sequences. PCR amplification was performed using as template both ligation products (50 μl PCR volume containing 10 μl of the ligation reaction). Agarose gel electrophoresis was used to check MLPA efficiency. Then, PCR reaction fragments were separated by capillary gel electrophoresis (ABI 3739, Applied Biosystems) and quantified using the Genemapper software (Applied Biosystems). MS-MLPA processing was performed using Coffalyser analysis tool developed at MRC-Holland (http://www.mlpa.com) and performed as described by Jeuken et al [32].

## Results

### Patients with higher number of CD133+ cells could present resistance to the treatment

**Figure 2:**
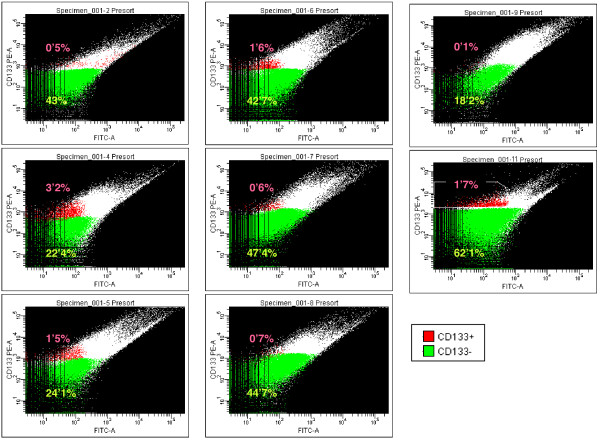
**FACS sorting of GBM cells using CD133 antibody**. Dot plot representation of CD133+ and CD133- populations in GBM tumour samples are shown. CD133+ population is painted in red and CD133- population in green. Percentage of each population is marked (green for CD133- and red for CD133+ percentage). Tumour sample is illustrated in the upper side of each plot.

In order to check the functionality of the CD133 antibody and the FACS, we firstly tested the methodology in a human bone marrow sample. In normal conditions, CD133 antibody also identifies a subset of bone marrow stem cells, which are also CD34 positives. Figure 1 shows how CD133 antibody identifies a pool of well-defined human CD133+/CD34+ cells. Using the same procedure, we sorted the CD133 positive population from each fresh GBM sample without previous cell culture (Figure 2). Table 1 shows the absolute number and percentage of CD133+ cells obtained from each sample as well as clinical-biological parameters of each patient. It is interesting to note that only those two patients with higher number of CD133+ cells (more than 10000 cells) did not response to the chemotherapy. Interestingly this correlation, high CD133+ cell number and resistance to therapy, has also been observed in patients and in GBM cultured cells [33-35] which further validate our approach.

### *EGFR*, *PTEN *and *MGMT *genes are altered in the GBM primary samples

**Figure 3:**
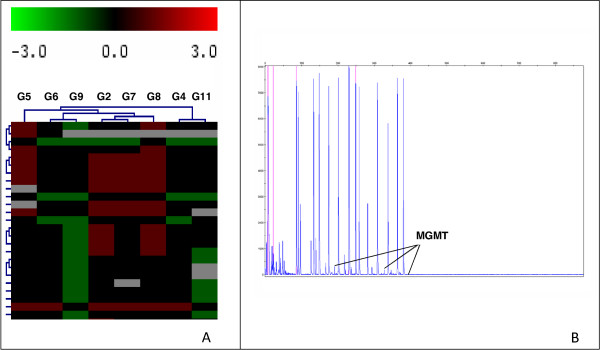
CGH array and MGMT promoter methylation assays in GBM samples.** A)** Unsupervised cluster analysis of CGH data from 8 primary GBMs. Each column represents one case and each row represents one BAC clone. We assigned values of 1, 0 and -1 for gain, no change and loss, respectively. Losses are in green and gains in red. P-values < 0.05. **B)** Ideogram showing MGMT promoter methylation.

The implication of different genetic alterations in the prognosis of primary glioblastoma such as *EGFR *amplification, *PTEN *deletion or *MGMT *promoter methylation has been previously described. As *EGFR*, *PTEN *and *MGMT *genes are usually altered in primary GBMs, we decided to corroborate the primary nature of our samples by checking for the existence of these alterations in the bulk tumour cell population of GBMs by FISH analysis and MLPA assay. *EGFR *amplifications were detected in 3 of 8 samples and *PTEN *deletions in 6 of 8 samples. Additionally, we also detected *MGMT *promoter methylation but none of these variables were significantly related to the biological features of the patients (see Table 1 and Figure 3B). It is important to remark that the number of cases studied is probably too low to find this kind of correlation.

### Common genomic imbalances identify patients with higher number of CD133+ cells without treatment response

In addition to the expression studies, we analyzed the genomic profile by CGH in the bulk tumours. All P-values reported were two-sided and statistical significance was defined as P-values < 0.05. In more than 50% of cases the gains affected to chromosomal regions located at 1q31-q42, 3q25, 4p15, 6p21-6q23, 7p21, 7q21, 9p21, 9q22, 11q22, 12q21 and 18q12. The losses were located on regions at 1p36, 1p13, 2p23, 5p15, 10q24.16, 12q13, 13q14, and 17p were also affected on more than 50% of cases [Additional file 4: Supplemental table S2]. Two major genetic groups emerge from the unsupervised clustering of CGH data (Figure 3A). Significantly, the only two cases with higher number of CD133+ cells and without treatment response, samples G4 and G11, grouped together. This cluster was characterized with common genomic rearrangements with gains on 3p21.31, 6p21, 6q25, 7p14.2, 9q22, 15q11, 20q13 and 22q13 chromosomes.

### CD133+ vs. CD133- gene expression analysis divided GBMs in 2 different groups

Although the number of samples is not very large, the main feature that distinguishes this work from previous studies [36,37] resides on the *ex-vivo *study of GBM cells that were analysed. Using direct sorting of CD133+ cells without previous cell culture led us to obtain a bona fide primary pool of CD133+ cells. Even when these cells represent a low percentage in the number of tumour cells, as in a normal tissue, we were able to isolate and amplify their RNA (by two rounds of amplification) in order to study their gene expression signature in comparison to its counterpart population of CD133- from the same GBM tumour.

Data normalized from preliminary analysis was used to calculate only those probe sets that were present (as described in the MAS5 algorithm) in all the samples (16 arrays from 8 GBM; hybridization per cell population). Results from this expression analysis have been deposited at GEO [25] with accession number GSE18015.

**Figure 4:**
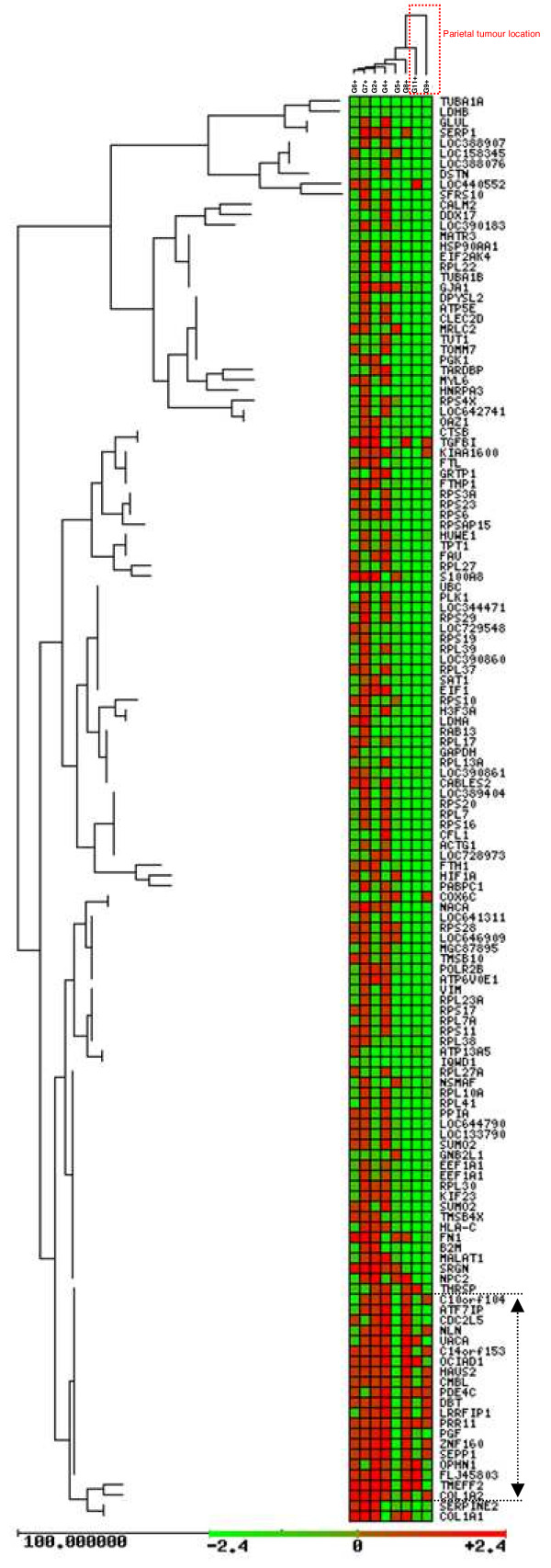
**Unsupervised clustering of CD133+ cells vs. CD133- cell gene expression signature from each tumour sample show 2 main GBM groups**. To molecularly characterize glioblastoma stem cells of GBM tumours, we compared the gene expression profiles of purified CD133+ cells from GBM patients versus CD133-cells from each patient. Each gene (identified at right) is represented by a single row of coloured boxes; each patient is represented by one single column. Data are displayed by a colour code where red indicates over-expression in CD133+ fraction versus CD133-cells. A group of genes over-expressed for almost all samples are grouped in the bottom. SOTArray tool from GEPAS Release v3.1, let us to classify CD133+ vs. CD133- cells from each tumour in 2 mainly groups: G9, G11 and the rest.

A final list of 245 probe sets was obtained according to these parameters. Initially, unsupervised clustering of 245 gene list using GEPAS Release v3.1 software (http://gepas3.bioinfo.cipf.es/) allowed us to examine the first classification of these GBMs. Importantly, GBM samples were ordered in two main groups (Figure 4). Samples G9 and G11 were grouped together and apart from the rest. However, their only common biological characteristic was their tumour location. G9 and G11 presented a parietal location versus the temporal or local locations presented by the remaining tumours (Table 1).

### Commonly up-regulated and down-regulated genes in CD133+ GBMs

**Figure 5:**
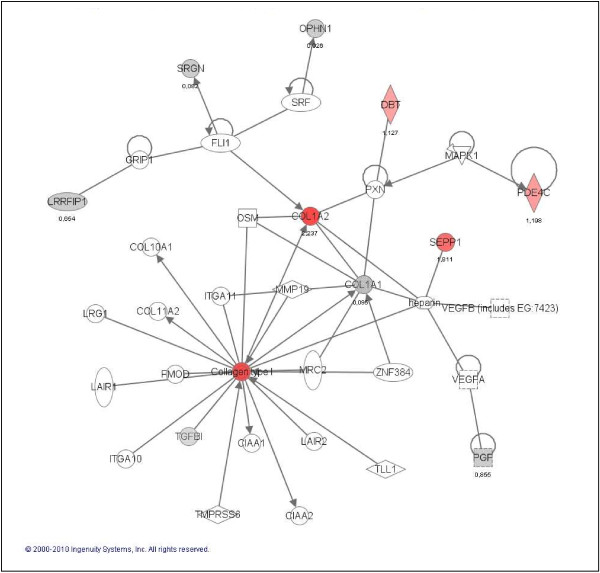
**Commonly CD133+ cell up-regulated genes participate in angiogenesis, tumour development and neural developmental disorders**. Ingenuity representation and classification by functions of those common up-regulated genes in all CD133+ vs. CD133- cell GBM samples. Red colour genes are the most positive deregulated and grey one those with a lower over-expression levels in this group. The first cluster of genes (*COL1A1, COL1A2, TGFB1*...) has been described largely in angiogenesis and permeability whereas the second cluster (*LRRFIP1 *and *OPHN1) *participates in developmental disorders. Changing transcription pattern of all of them favour tumour development.

**Figure 6:**
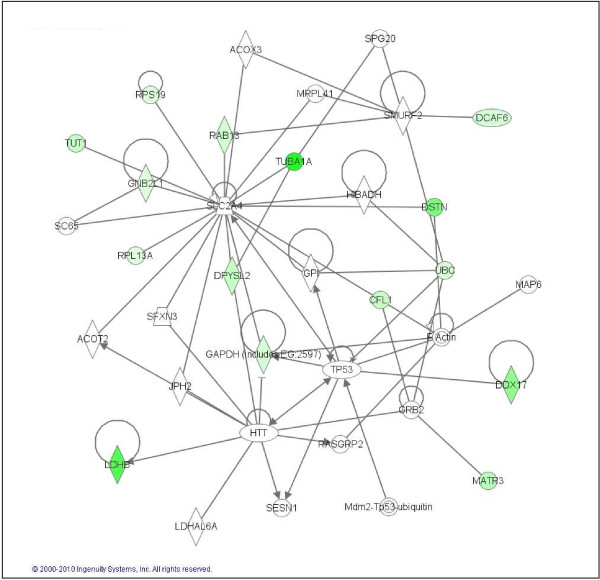
**Common CD133+ cell down-regulated genes are involved in cell assembly organization and cancer**. Ingenuity representation and classification by functions of those commonly down-regulated genes in all CD133+ vs. CD133- cell GBM samples. Green colour represents those genes differentially regulated in CD133+ vs. CD133- that participates in cell assembly, migration and cancer pathways.

Following the initial classification proposed by SOTArray of GEPAS, we were able to discriminate a minor group of genes commonly up (19 genes) and down-regulated (22 genes) in all samples (CD133+/CD133-) (Tables 2 and 3). Common up-regulation of genes such as *COL1A1*, *COL1A2*, *PGF, LRRFIP1, TMEFF2 *or *TGFB1 *suggested an important role of this pool of cells in blood vessel formation, angiogenesis, permeability and proliferation pathways, essentials functions in tumour progression [38-40] (Figure 5). On the other hand, the group of genes commonly down-regulated in all CD133+ vs. CD133- cells (that means, over-expressed in the CD133- compartment) were strikingly related to cell assembly, neural cell organization and molecular pathways altered in neurological disorders. That is the case of, *GNB2L1*, *DPYSL2*, *TUBA1A *or *CFL1*, all of them important players in cell migration, morphology and actin polymerization, in brief, motility of neural differentiated cells (Figure 6).

**Table 2:** Common up-regulated genes in CD133+ vs.CD133- GBM cells.

Gene symbol	Gene name	Probe set
COL1A1	Collagen, type I, alpha 1	1556499_s_at
TGFBI	Transforming growth factor, beta-induced, 68 kDa	201506_at
C10orf104	Chromosome 10 open reading frame 104	224667_x_at
UACA	Uveal autoantigen with coiled-coil domains and ankyrin repeats	236715_x_at
C14orf153	Chromosome 14 open reading frame 153	232814_x_at
OCIAD1	OCIA domain containing 1	239748_x_at
CMBL	Carboxymethylenebutenolidase homolog (Pseudomonas)	234981_x_at
PDE4C	Phosphodiesterase 4C, cAMP-specific (phosphodiesterase E1 dunce homolog, Drosophila)	206792_x_at
DBT	Dihydrolipoamide branched chain transacylase E2	205370_x_at
LRRFIP1	Leucine rich repeat (in FLII) interacting protein 1	211452_x_at
PRR11	Proline rich 11	219392_x_at
PGF	Placental growth factor, vascular endothelial growth factor-related protein	215179_x_at
ZNF160	Zinc finger protein 160	214715_x_at
SEPP1	Selenoprotein P, plasma, 1	237475_x_at
OPHN1	Oligophrenin 1	206323_x_at
COL1A2	Collagen, type I, alpha 2	202403_s_at
FLJ45803	FLJ45803 protein	238701_x_at
TMEFF2	Transmembrane protein with EGF-like and two follistatin-like domains 2	224321_at
SRGN	Serglycin	201859_at

**Table 3:** Common down-regulated genes in CD133+ vs.CD133- GBM cells.

Gene symbol	Gene name	Probe set
HNRPA3	Heterogeneous nuclear ribonucleoprotein A3	1555653_at
ATP13A5	ATPase type 13A5	1553567_s_at
IQWD1	IQ motif and WD repeats 1	224373_s_at; 224372_at
TUBA1A	Tubulin, alpha 1a	209118_s_at
DPYSL2	Dihydropyrimidinase-like 2	200762_at
RAB13	RAB13, member RAS oncogene family	202252_at
MATR3	Matrin 3	214363_s_at
DSTN	Destrin (actin depolymerizing factor)	201022_s_at
LDHB	Lactate dehydrogenase B	213564_x_at
UBC	Ubiquitin C	211296_x_at
CFL1	Cofilin 1 (non-muscle)	200021_at
LOC729548	Similar to ribosomal protein S2	203107_x_at
GAPDH	Glyceraldehyde-3-phosphate dehydrogenase	213453_x_at; 212581_x_at; 217398_x_at
LOC388076	Similar to ribosomal protein S8	200858_s_at
RPS19	Ribosomal protein S19	202649_x_at; 213414_s_at
TUT1	Terminal uridylyl transferase 1, U6 snRNA-specific	200689_x_at; 211345_x_at; 211927_x_at
RPSAP15	Ribosomal protein SA pseudogene 15	213801_x_at
LOC390860	Similar to 60S acidic ribosomal protein P0 (L10E)	211720_x_at; 208856_x_at; 201033_x_at
DDX17	DEAD (Asp-Glu-Ala-Asp) box polypeptide 17	208718_at
RPL13A	Ribosomal protein L13a	212790_x_at
GNB2L1	Guanine nucleotide binding protein (G protein), beta polypeptide 2-like 1	200651_at
LOC390861	Similar to cytoplasmic beta-actin	200801_x_at

### Two different GBM groups can be functionally defined according to the expression pattern of 40 genes

**Figure 7:**
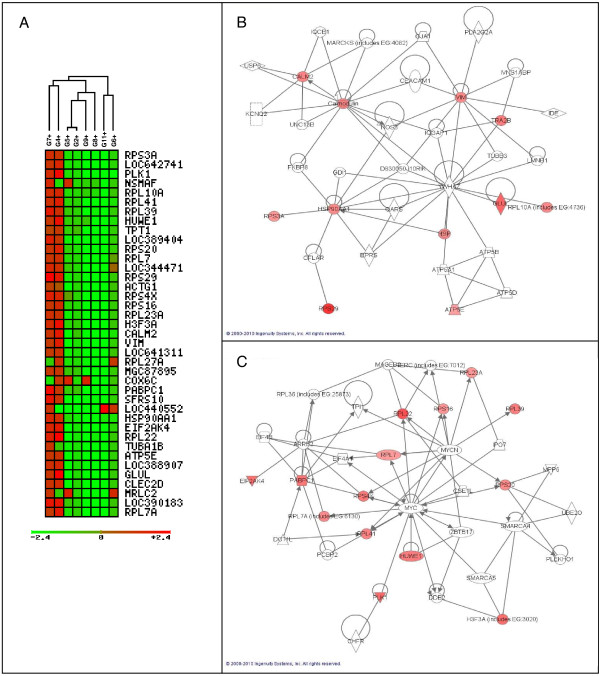
**Forty differential genes in G4 and G7 samples discriminate between high or low proliferative potential**. Unsupervised **c**lustering and ingenuity pathways representation of 40 differentially expressed genes. **A)** Unsupervised clustering of this 40 gene list let us to distinguish 2 well defined and opposite groups. Ingenuity principal represented pathways include **B)** recombination and repair pathways and **C)** cancer and cell compromise. Those GBMs with a positive pattern CD133+/CD133- for this gene expression signature, could present a higher proliferative potential of their tumour stem cells or, by the opposite, a lower proliferative potential of the mature glioma cells.

**Figure 8:**
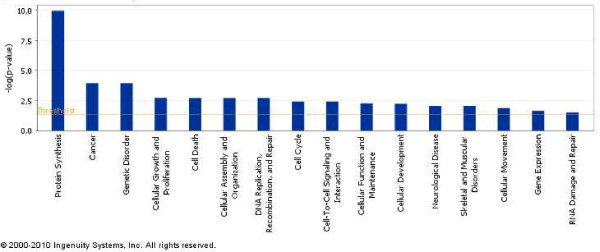
**Forty differentially expressed genes in ex-vivo CD133+/CD133- GBM cells classify these tumours according to their functional categories**. Ingenuity functional classification of 40 differentially expressed genes in primary GBMs discriminate two main groups of GBM according to their proliferative potential.

The major group of genes discriminated by the SOTArray of GEPAS presented a differential gene expression pattern in two of the eight CD133+ vs. CD133- GBM samples, G4 and G7, in contrast to the remaining cases. In this group, we found out a cluster of genes clearly over-expressed in some of CD133+ vs. CD133- GBM samples (G4 and G7) but repressed in the rest. Specifically, a group of 40 well-defined genes, classified according to their function, were able to distinguish between 2 different GBM signatures (Figure 7) revealing the possible different proliferative potential in high grade GBM tumours (*VIM*, *GLUL*, *PLK1*, *HUWE1, RPS4X*...) (Figure 8 and Table 4).

## Discussion

Glioblastomas are the higher-grade gliomas with fast progression and unfortunate prognosis. Recent studies have demonstrated in these tumours the existence of a small fraction of cancer cells endowed with features of primitive neural progenitor. Although some observations pointed out towards the involvement of CD133- cells in tumour maintenance [24], several studies have involved the CD133^+ ^cells as the brain tumour initiating cell [6,41,42]. In any case, studies performed in order to characterize the glioblastoma stem cell have been carried out using *in vitro*, cultured tumour cells. While these cultured cells present the capacity to form NS, essential pathways in cell/tumour biology could likely be altered as a direct consequence of the cell culturing such as cell-cell adhesion, cell-niche junctions, exposure to mitogen activation, rapid division of the cells etc.

To gain insight into the characterization of these cells, we examined directly for the first time CD133+ cells by FACS-based purification from *ex-vivo *primary tumours without the intervention of cell culturing or any prior expansion procedure.

Despite that the cohort of tumours analysed was not very large, we could find a correlation among clinical history, response to treatment and genomic alterations in two samples (G4 and G11). Both of these tumours showed the highest content of CD133+ cells, the lack of response to treatment and similar chromosomal alterations (multidimensional scaling analysis reflected relationship among these parameters). However, this correlation was not further supported by transcription profiling of CD133+ and CD133- cells. Indeed, non-supervised analysis of CD133+ vs. CD133- gene expression (Figure 4) showed that only G9 and G11 samples were grouped together and apart from the rest, being the tumour location the only biological feature able to distinguish them (see Table 1). Despite of this low number of samples, multidimensional scaling analysis established again a relationship between G9/G11 tumour location and their gene expression.

To understand the biological properties of the CD133+ compartment, we sought to identify common gene signature by the comparison of CD133+ vs. CD133- cell populations. This array-based analysis led us to the identification of gene profiles with common up-regulated and down-regulated genes. Up-regulated genes such as *COL1A1*, *COL1A2*, *PGF *[38] or *TGFB1 *[43], suggested an important role of these compartment in blood vessel formation, angiogenesis, permeability and invasiveness, essential functions in tumour progression [38-40]. Significantly, most of these up-regulated genes encoded secreted proteins involved in autocrine and paracrine signalling, like *TGFBI*, a pleiotropic cytokine that, among other functions, can induce the dissociation of VE-cadherin junctions between endothelial cells which could favour mature tumour or GBM cells migration [43]. Up-regulation of these genes in putative CD133+ stem cells would help to increase the mobility of cancer stem cells through the brain, which is consistent with the high invasive characteristics of these tumours and their high possibility to colonize the adjacent area. It is also worthy to mention in this same regard, the importance of the microenvironment in the stem cell/cancer stem cell maintenance, as has recently been pointed out with the identification of the perivascular niche in grade I-IV astrocytomas [44]. Several evidences suggest that normal neural stem cells, and likely also neural cancer stem cells, exist within protective niches as the vascular niches, into which endothelial cells secrete factors that regulate neural stem cell function [45,46]. This raises the question of whether CSCs could be located and regulated by these microenvironments. Calabrese et al. proposed that the tumour microvasculature generates specific niche microenvironments promoting the maintenance of CSCs [47]. Recent studies using orthotopic glioblastoma xenografts suggest that CSCs secrete proangiogenic factors that promote the recruitment and formation of tumour blood vessels [48] that significantly facilitates brain tumour growth and invasion. Our gene expression findings in *ex-vivo *CD133+ isolated cells clearly support this result.

High expression in the CD133+ compartment of genes such as *LRRFIP1*, transcriptional repressor of *EGFR *[49], would support the idea of *EGFR *gene as a secondary event in the process of GBM development by promoting infiltration and mediating resistance to therapy. In this same scenario, the positive regulation of the tumour suppressor gene *TMEFF2 *[50] in the potential CD133+ stem cell compartment in these GBMs, could also operate as a late event in the initiation of neoplastic progression. In fact, low levels of *TMEFF2 *and other genes responsible for tissue or cell assembly in the CD133- compartment would promote the down-regulation of cell to cell interactions and junctions, providing a molecular mechanism for the highly invasive nature of the GBM.

The second group of genes, commonly down-regulated in all CD133+ vs. CD133- cell from human *ex-vivo *GBM samples (that means, over-expressed in the CD133- compartment) were found to be associated to cell assembly, neural cell organization and neurological disorders. That is the case of genes such as *GNB2L1*, an anchor protein involved in adhesion and migration of human glioma cells [51], *DPYSL2*, a promoter of microtubule assembly and neuronal development [52], *TUBA1A *[53] or *CFL*, which controls cell migration and cell cycle progression [54,55]. This group of genes plays important roles in cell migration, cell polarity and actin polymerization (Figure 6). In this same oncogenic scenario, it would be interesting to mention the deregulated expression of *HIF-1 *gene. This gene which is down-regulated in most of the CD133+ samples analysed, is involved in tumour angiogenesis and cell growth [56], and could play some role in the later events that drive tumour progression. In this regard, recent studies have demonstrated that HIF-1 protein stabilization contribute to tumour angiogenesis, one of the main characteristics of primary GBMs [16]. Mutations in metabolic enzymes, in particular isocitrate dehydrogenase enzymes (IDH1 and IDH2), have been shown to be involved in glioma development and would facilitate HIF-1 protein stabilization [57,58]. The negative deregulation of the *HIF-1 *gene that we have observed in most *ex-vivo *CD133+ cells in this work, also support this idea.

A notable feature of the gene expression pattern of CD133+ cells was the differential expression of 40 genes that divide GBM samples in two opposite molecular signatures. The classification of these 40 genes according to their function (Figure 8 and Table 4) pointed to their implication in cell growth, cell death, DNA replication, recombination and, definitively, in cell proliferative control. Amongst these genes we wanted to emphasize the differential expression of the gene coding for vimentin (*VIM)*, an intermediate filament of the mesenchymal lineage involved in migration, cell signalling, cancer and neurological disease [59,60]. Some other genes differentially expressed in this pool of cells CD133+ and also involved in cancer and neurological disease are *RPS4X*, *RPS3A *or *TUBA1B *(Table 4). Another relevant member of the top 40 list of genes was *HUWE1*, a pleitropic ubiquitin ligase that participates in a wide variety of biological functions related to cell proliferation such as cell growth/death, and DNA replication, and that has been described to be deregulated in different carcinomas [61] (see Figure 8 and Table 4). Interestingly, this deregulated gene has also been reported to be an important control gene for the proliferation capacity of embryonic NSC in the mice [62]. *GLUL *encodes the glutamine synthetase, a metabolic enzyme required for the maintenance of the energy balance and that when mutated causes severe malformations and neonatal death [63]. Finally, *PLK1*, the mitotic kinase par excellence, modulates mitosis entry and promotes cell transformation upon upregulation as an oncogene [64-66]. These differentially regulated molecules must be playing pivotal roles in keeping the tumour cells in a switch-on state that enables them to survive, proliferate and invade the surrounding healthy tissue.

**Table 4:** Functional classification of 40 differentially expressed genes in CD133+ vs.CD133- GBM samples

CATEGORY	P-VALUE	MOLECULES
Protein Synthesis	1,14E-10-1,14E-10	EIF2AK4, RPL22, RPS4X, RPS3A, RPL27A, RPL7A, RPL39, RPL23A, RPL41, RPL7
Cancer	1,17E-04-4,38E-02	HUWE1, TRA2B, VIM, PLK1, TUBA1B, ACTG1, RPL7, TPT1, RPS4X, RPS3A, H3F3A, RPS16, GLUL, HSP90AA1, CLEC2D
Cellular Growth and Proliferation	1,94E-03-2,89E-02	HUWE1, PLK1, RPL23A
Cell Death	1,99E-03-3,52E-02	HUWE1, RPS3A, HSP90AA1, VIM, PLK1
Cell Morphology	1,99E-03-2,36E-02	VIM
Cellular Assembly and Organization	1,99E-03-3,71E-02	VIM, PLK1, ACTG1, RPL7
DNA Replication, Recombination, and Repair	1,99E-03-3,71E-02	HUWE1, VIM, PLK1
Cell-To-Cell Signalling and Interaction	3,97E-03-9,9E-03	VIM
Cellular Function and Maintenance	5,49E-03-2,94E-02	EIF2AK4, HSP90AA1, VIM, ACTG1
Cellular Development	5,95E-03-1,97E-02	EIF2AK4, HSP90AA1, VIM
Neurological Disease	9,02E-03-4,28E-02	TPT1, RPS4X, RPS3A, RPL39, VIM, ACTG1, NSMAF, TUBA1B, CALM2
Skeletal and Muscular Disorders	9,02E-03-9,02E-03	TPT1, RPS4X, RPS3A, VIM, TUBA1B
Cellular Movement	1,38E-02-1,38E-02	VIM
Gene Expression	2,36E-02-3,13E-02	PABPC1, PLK1

In brief, the results obtained in this study revealed the presence in CD133+ cells from primary glioblastoma of a common gene expression signature involved principally in the promotion of proangiogenic and invasive programs. Additionally, CD133+ gene expression pattern led us to discriminate between two different GBM subtypes in higher or lower proliferative tumours. The molecular biology and the expression signature of these CD133+ cells that drive and support the tumour growth will shed light on the development of new treatments to fight against GBMs.

## Conclusions

*Ex-vivo *analysis of CD133+ primary GBM cells has driven us to the valid detection of a common gene expression profile among GBMs principally characterized by the expression of genes involved in blood vessel formation, angiogenesis and invasiveness, the main aspects of glioma biology that contributes to its adverse prognosis. Besides, data obtained from the analysis of a group of 40 genes differentially expressed in GBM samples suggest that primary GBM can be sub-classified according to the properties of their CD133+ cells. Differences between both groups were provided by the proliferative potential of their CD133+ population (potential tumour stem cells). We can conclude that molecular characterization of CD133+ population in primary GBMs could be critical in the development of new and effective treatments for these tumours with very dismal prognosis.

## Abbreviations

GBM: Glioblastoma multiforme; FACS: Fluorescent activated cell sorting; CGH: Compared genomic hybridization; FISH: Fluorescence *in situ *hybridization; MLPA: multiplex ligation-dependent probe amplification; CSCs: Cancer stem cells; MACS: Magnetic activated cell sorting; NS: Neurospheres; NSCs: Neural stem cells; BM: bone marrow; BAC: bacterial artificial chromosome.

## Competing interests

The authors declare that they have no competing interests.

## Authors' contributions

JLG participated carrying out CGH, FISH and methylation studies. MPC carried out qPCR assays, analysis and interpretation of array data and has been involved in drafting the manuscript. JAGM provided GBM samples and clinical data from patients. FGR carried out gene expression assays and performed gene expression analysis. JO, OB and MS contributed to anatomy-pathological diagnosis of GBM samples. JMHR and RGS have been involved in revising it critically for important intellectual content. MSM conceived of the study, its design, coordination and has been involved in drafting the manuscript. All authors read and approved the final manuscript.

## Pre-publication history

The pre-publication history for this paper can be accessed here:

http://www.biomedcentral.com/1471-2407/10/454/prepub

## Supplementary Material

Additional file 1 **Complementary details on CGH array method**. Complementary details of the CGH methodology in GBM tumours. Click here for file

Additional file 2 **qPCR Primer Sets**. Set of primers carefully designed to test mRNA expression in CD133+ and CD133- cells by SYBR Green real-time PCR. Click here for file

Additional file 3 **qPCR validation of gene candidates differentially expressed in Affymetrix arrays**. Relative expression of six gene candidates differentially expressed between CD133+ cells and CD133- cells from four representative groups of samples is shown. Names of transcripts analyzed are on the x-axis and the CD133+/CD133- mean fold differential regulation is on the y-axis. Click here for file

Additional file 4 **CGH-array data set**. Chromosomal gains and losses from tumour bulk cells of GBM patients are shown in this table. Each column shows the median of Cy5/Cy3 ratio intensity of triplicate spots of each clone normalized by GEPAS. Click here for file
