# Supplementary material for: Molecular analysis of ex-vivo CD133+ GBM cells revealed a common invasive and angiogenic profile but different proliferative signatures among high grade gliomas
Source: BMC Cancer. 2010 Aug 24;10:454. doi: 10.1186/1471-2407-10-454 (PMC2939550; doi:10.1186/1471-2407-10-454)
Supplement: Additional file 1 — Complementary details on CGH array method. Complementary details of the CGH methodology in GBM tumours. [file 1471-2407-10-454-S1.DOC]

**Additional file 1- Complementary details on CGH array method.**

The DNA from each fresh-frozen sample was extracted with the standard phenol-chloroform method and reference DNA was prepared from human placenta of healthy donors. All DNAs were quantified using the Nanodrop spectrophotometer. 10 ng of DNA (BAC/PAC) was used as a template for three DOP-PCRs. These products were ethanol precipitated and dissolved in distilled water. A minimum of three replicates per clone were printed by *Microgrid II* (Biorobotics) on each slide (*Ultragaps Coated Slides*, Corning) using aqueous DMSO buffer as spotting solution. Briefly, for labelling reactions, 3 μg of non amplified genomic DNA, test (DNA of tumour) and reference (DNA of placenta) were digested separately with DpnII restriction enzyme (New England Biolabs, Beverly, MA). For microarray hybridization, the digested DNAs were separately labelled using random primers (Bioprimer labelling kit, Invitrogen) and Cy3-dCTP and Cy5-dCTP fluorescent dye to paired hybridization samples (Amersham Biosciences). The incorporation of the label nucleotide was quantified using the Nanodrop spectrophotometer. Labelled test and reference DNAs were mixed equitably, co-precipitated in presence of Cot-1 human DNA (Roche, Indianapolis, IN) with ethanol, washed, and resuspended in hybridization solution (50% Formamide, 10% Dextran sulfate, 2X standard saline citrate, 10 mM Tris pH 7.6, 2.7% sodium dodecyl sulfate and 10 μg/ μl of yeast tRNA). DNA mixtures were co-hybridized to the arrays in the GENETAC for 48 hours at 37ºC according to the manufacturer’s recommended protocol. Images and signal intensities were acquired using GenePix4000B (Axon Instruments, Burlingame, CA) dual laser scanner in combination with GenePixPro4.0 (Axon Instruments) imaging software. For the array, 10 simultaneous hybridizations of normal male versus normal female and placenta (DNA reference) was performed to define the normal variation for the log_2_ ratio.

The Cy5/Cy3 intensity ratios of every spot were converted into log_2_ ratios. The log_2_ ratio of each clone was normalized to the median log_2_ ratio of the ten control hybridizations, after which the median of triplicate spots was calculated. Data from two-colour hybridizations for both DNAs were normalized using the corresponding GEPAS module DNMAD. Regions of copy number gained and lost for the BAC array-CGH data were identified by creating sample specific thresholds. The clones with log2 ratios up or down control sample’s threshold value (0.4) were considered gains or losses, respectively. A BAC clone with a log_2_ ratio of -0.4 or less was defined as a loss region and log_2_ ratio of +0.4 or more was defined as a gain region. Moreover, spots with weak Cy3 or Cy5 below R^2^ < 0.2 intensity were excluded. Furthermore, clones with standard deviation more than 0.3 of triplicate spots were also excluded. In total, approximately 10% of clones were excluded.
