# Supplementary figures and images for: Molecular analysis of ex-vivo CD133+ GBM cells revealed a common invasive and angiogenic profile but different proliferative signatures among high grade gliomas
Source: BMC Cancer. 2010 Aug 24;10:454. doi: 10.1186/1471-2407-10-454 (PMC2939550; doi:10.1186/1471-2407-10-454)

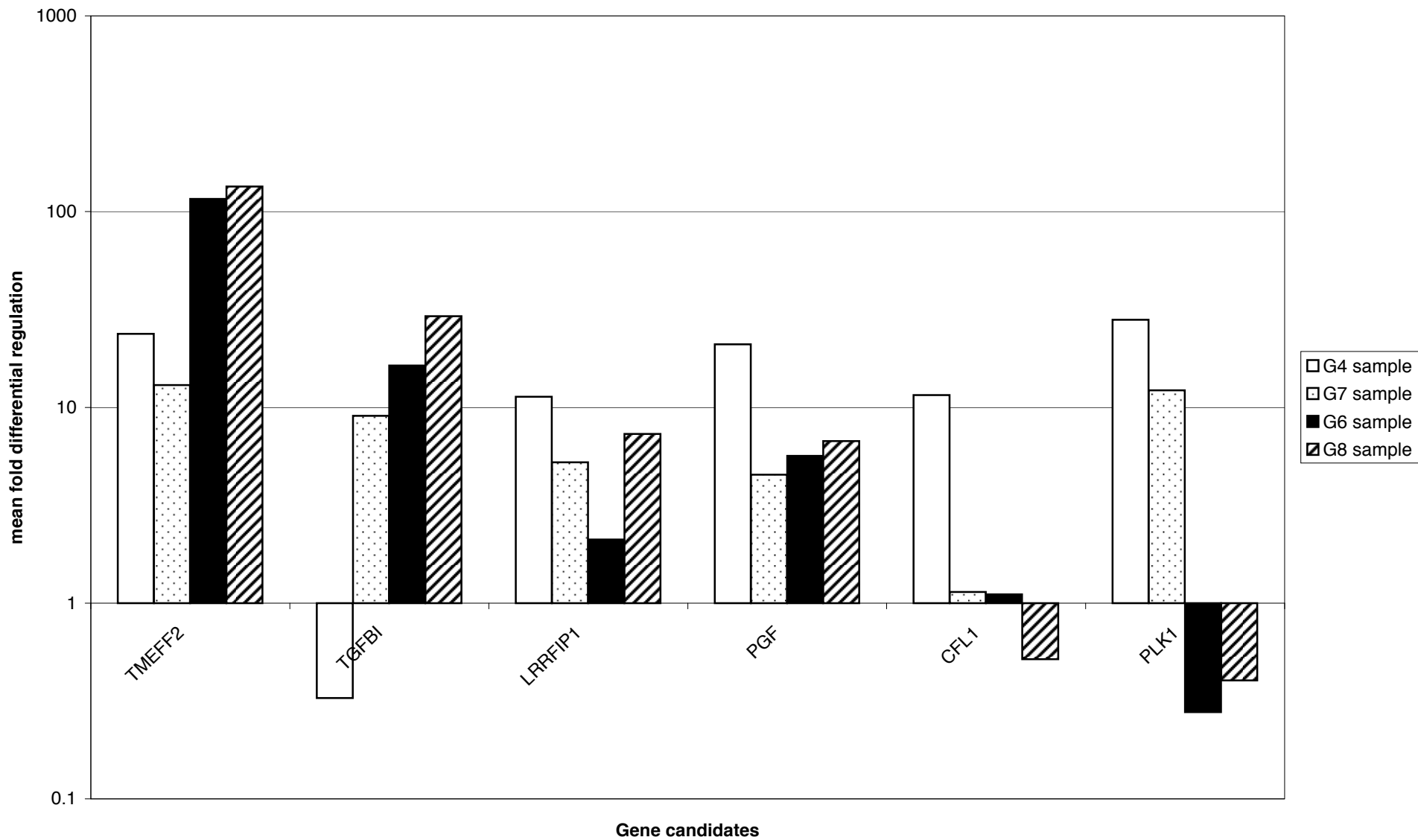

Supplement: Additional file 3 — qPCR validation of gene candidates differentially expressed in Affymetrix arrays. Relative expression of six gene candidates differentially expressed between CD133+ cells and CD133- cells from four representative groups of samples is shown. Names of transcripts analyzed are on the x-axis and the CD133+/CD133- mean fold differential regulation is on the y-axis. [file 1471-2407-10-454-S3.PDF]
